# Enhanced Specificity and Drug Delivery in Tumors by cRGD - Anchoring Thermosensitive Liposomes

**DOI:** 10.1007/s11095-015-1746-7

**Published:** 2015-07-23

**Authors:** Bilyana M. Dicheva, Timo L. M. ten Hagen, Ann L. B. Seynhaeve, Mohamadreza Amin, Alexander M. M. Eggermont, Gerben A. Koning

**Affiliations:** Laboratory Experimental Surgical Oncology, Section Surgical Oncology Department of Surgery, Erasmus MC Cancer Center, Rotterdam, The Netherlands; Institut Gustave Roussy, Villejuif, France; Department of Pharmaceutics, Mashhad University of Medical Sciences, Mashhad, Iran; Laboratory of Experimental Surgical Oncology, Department of Surgical Oncology, Erasmus MC Cancer Institute, PO Box 2040, 3000 CA Rotterdam, The Netherlands

**Keywords:** Doxorubicin, drug delivery, hyperthermia, RGD - thermosensitive liposomes

## Abstract

**Purpose:**

To develop RGD-targeted thermosensitive liposomes with increased tumor retention, improving drug release efficiency upon mild hyperthermia (HT) in both tumor and angiogenic endothelial cells.

**Methods:**

Standard termosensitive liposomes (TSL) and TSL containing a cyclic Arg-Gly-Asp (cRGD) pentapeptide with the sequence Arg-Cys-D-Phe-Asp-Gly (RGDf[N-Met]C) were synthetized, loaded with Dox and characterized. Temperature- and time-dependent drug release profiles were assessed by fluorometry. Intracellular Dox delivery was studied by flow cytometry and confocal microscopy. Cytotoxic effect of TSL and RGD-TSL was studied on B16Bl6 melanoma, B16F10 melanoma and HUVEC. Intravital microscopy was performed on B16Bl6 tumors implanted in dorsal-skin fold window-bearing mice. Pharmacokinetic and biodistribution of Dox-TSL and Dox-RGD-TSL were followed in B16Bl6 tumor bearing mice upon normothermia or initial hyperthermia conditions.

**Results:**

DLS and cryo-TEM revealed particle homogeneity and size of around 85 nm. Doxorubicin loading efficiency was >95%as assessed by spectrofluorometry. Flow cytometry and confocal microscopy showed a specific uptake of RGD-TSL by melanoma and endothelial cells when compared to TSL and an increased doxorubicin delivery. High resolution intravital microscopy demonstrated specific accumulation of RGD-TSL to the tumor vasculature. Moreover, application of hyperthermia resulted in massive drug release from RGD-TSL. Biodistribution studies showed that initial hyperthermia increases Dox uptake in tumors from TSL and RGD-TSL.

**Conclusion:**

RGD-TSL have potency to increase drug efficacy due to higher uptake by tumor and angiogenic endothelial cells in combination with heat-triggered drug release.

**Electronic supplementary material:**

The online version of this article (doi:10.1007/s11095-015-1746-7) contains supplementary material, which is available to authorized users.

## Introduction

Nanoparticles, such as liposomes have passed different stages of modifications in their design and nowadays are commonly used in cancer chemotherapy (1). Stealth liposomal nanoparticles of 100 nm are believed to accumulate passively in the tumor due to the leaky tumor vasculature and related enhanced permeability and retention effect (2, 3). Liposomes have contributed significantly to decrease toxic side effects caused by free drug administration (4–6). However, tumor accumulation of anticancer drugs using liposomes seemed far from optimal to guarantee improvement in therapeutic efficacy in clinical practice (4, 7). Low specificity of liposomes and their high intrinsic stability limit therapeutic outcome. Selectivity and efficacy can be achieved by decorating liposomes with targeting ligands, while an external trigger, e.g., heat, can control liposomal drug release. In this study we aimed at developing RGD-targeted thermosentitive liposomes, which combine active targeting of tumors together with a heat-triggered drug release function. These nanoparticles contain multiple RGD peptides on their surface to achieve tumor specificity and increased retention in tumors and a thermosensitive bilayer for heat-triggered drug release.

In order to efficiently target tumor cells, liposomes first need to extravasate from tumor vasculature and penetrate the tumor tissue. However, the extravasation process is usually heterogeneous and inefficient (3, 8). Targeting of tumor vasculature rather than tumor cells has become a promising approach in cancer therapy (9). In this case several tumor pathophysiological barriers do not play a role as endothelial cells are easily accessible to circulating chemotherapeutic drugs and deep penetration into tumor tissue is not necessary. Moreover, destruction of tumor vessels leads to indirect killing of tumor cells, which depend on their supply of oxygen and nutrients. Finally, endothelial cells are genetically stable and therefore less resistant to drug therapy (10). Besides vascular targeting, those small liposomes may extravasate through the leaky tumor vasculature and target tumor cells in addition (11).

Various vascular targets have been studied for anti-vascular therapy of which αvβ3 integrins have been used most often (12, 13). These integrins have been found to be overexpressed on tumor vasculature, but also on some metastatic melanoma cells (14, 15). The RGD (Arg-Gly-Asp) sequence is known to be a recognition motif for integrins such as αvβ3 (16). Binding to either tumor or endothelial cells via specific receptors may lead to internalization of liposomal chemotherapy thereby bringing the drug closer to the nucleus. The combination of targeting properties of this nanoparticle with the heat-triggered release function might aid at releasing the drug locally in the tumor. Besides triggering drug release, hyperthermia (HT) is known to play a role in changing tumor environment by increasing tumor blood flow, oxygenation and vessel permeability (16–21).

An abundant amount of literature is available on thermosensitive or targeted liposomes (22–25). However, the combination of both strategies in one carrier is a promising approach. In the present study we describe the design, characterization and behavior of RGD-targeted thermosensitive liposomes containing the chemotherapeutic drug doxorubicin *in vitro* and *in vivo*. These liposomes were decorated with a novel and specific cyclic Arg-Gly-Asp (cRGD) pentapeptide containing the sequence Arg-Cys-D-Phe-Asp-Gly (RGDf[N-Met]C) (26). We tested the cytotoxic effect of those liposomes on melanoma cell lines and endothelial cells. Extensive live cell imaging was performed to study their intracellular fate *in vitro. In vivo*, their affinity for tumors, drug release kinetics and uptake were studied in dorsal skin fold window chamber models implanted with melanoma B16Bl6 tumors. Pharmacokinetics and biodistribution of Dox encapsulated in either TSL or RGD-TSL were investigated in mice implanted with B16Bl6 tumors.

## Materials and Methods

### Chemicals

The phospholipids 1,2-dipalmitoyl-sn-glycero-3-phosphocholine (DPPC), 1,2-distearoyl-sn-glycero-3-phosphocholine (DSPC), 1,2-distearoyl-sn-glycero-3-phosphoethanolamine-N-PEG2000 (DSPE-PEG2000) were ordered from Lipoid (Ludwigshafen, Germany). 1,2-dipalmitoyl-sn-glycero-3-phosphoethanolamine-N-(7-nitro-2-1,3 benzoxadiazol-4-yl) (NBD-PE) and 1,2-Disteroyl-sn-Glycero-3-Phosphoethanolamine-N-[Maleimide (Polyethylene Glycol)2000] (Ammonium Salt) were purchased from Avanti Polar Lipid Inc. Doxorubicin-HCl was from Pharmachemie (Haarlem, The Netherlands). The RGDf[N-Met]Cys was provided by Peptron, South Korea. Sodium 3′-[(1-phenylaminocarbonyl)-3,4-tetrazolium]-bis(4-methoxy-6-nitro)benzene sulfonic acid hydrate (XTT) was purchased from Sigma-Aldrich (Zwijndrecht, The Netherlands). LysoTracker Red DND-99 and Dioctadecyl tetramethylindotricarbocyanine perchlorate (DiD-C_18_(3)) were purchased from Invitrogen.

### Preparation of TSL

TSL and RGD-TSL were composed of DPPC:DSPC:DSPE-PEG2000 in a molar ratio 70:25:5. RGD peptide was coupled to mPEG in a molar ratio 1.1:1 (peptide:lipid) and coupling efficiency was confirmed by MALDI analysis. For MALDI analysis, all peptide samples were prepared as 0.001 M in water. The matrix-assisted laser desorption/ionization (MALDI) 2,5-dihyfroxy benzoic acid was prepared as 10 mg/mL in water. Ten microliter of sample and 90 uL of matrix solution were mixed and 0.5 uL of this mixture was spotted on an anchor chip plate and allowed to dry at ambient temperatures. MALDI-TOF mass spectra were recorded on a Bruker Ultraflex III mass spectrometer, Bremen, Germany (27). Coupling of >95% was used for further liposome preparation. Liposomes were prepared by lipid film hydration and extrusion method (16). The lipids were dissolved in chlorophorm and methanol (9:1 vol/vol). TSL used for confocal microscopy contained 0.3 mol% of NBD-PE or 0.3 mol% DiD in the lipid bilayer. TSL used for intravital microscopy contained 0.3 mol% of DiD. The solvent was subsequently evaporated in a rotary evaporator until homogeneous lipid film was formed. The lipid film was hydrated in 250 mM (NH_4_)_2_SO_4_ solution with a pH 5,0 at 60°C for 30 min. The spontaneously formed liposomes were extruded subsequently 5 times through 100 nm, 5 times through 80 nm and 5 times through 50 nm polycarbonate filter (thermo barrel extruder at 60°C) and resulted in <100 nm TSL. (NH_4_)_2_SO_4_ outside of liposomes was removed from liposomal (NH_4_)_2_SO_4_ by a PD-10 to PD 10 (GE Healthcare, Buckinghamshire, UK), eluted with HEPES buffer, pH 7.4 (10 mM HEPES, 135 mM NaCl). Size and polydispersity index (PDI) were measured by dynamic light scattering using Zetasizer Nano ZS (Malvern Instruments, Worcestershire, UK). Lipid concentration was analysed by phosphate assay (27). After the phosphate concentration was determined, doxorubicin was loaded into the liposomes in 0,05:1 drug:lipid ratio (mol:mol) at 38°C for 1 h. The liposomes were concentrated by ultracentrifugation for 2 h, 4°C, 106.000 ×g (Ti50.2 rotor). The pellet was resuspended in HEPES buffer, pH 7.4 and left overnight on slow rotation at 4°C. Then the liposomes were passed through PD 10 column eluted with HEPES buffer, pH 7.4 to remove free doxorubicin. Doxorubicin concentration was measured by spectrophotometer at Ex 480 nm and loading efficiency (%) determined as [Dox/Lipid] after loading/[Dox/Lipid] before loading x100.

### TEM Cryo Imaging

Samples were prepared by adding a 2 μl droplet of liposome suspension to a lacy carbon film and subsequently plunge-freezing this sample into liquid ethane using a Vitrobot. An amorphous (‘vitrified’) ice film which contains the particles of interest was created. Cryo-TEM studies were performed using a FEI TECNAI F30ST (300 kV, using a cryo-holder, keeping the sample at –174°C during the studies). Imaging was done in low-dose mode on a CCD camera (image size 1 × 1 k).

### Stability at Physiological Conditions

Stability of TSL and RGD-TSL was established by incubating 10 mM [lipid]) in pre-heated FCS (1:149 *v/v*) under stirring and Dox release was measured for 1 h at 37°C. Samples without incubation were considered as a blank (Io). TSL were destroyed by adding 10% Triton X-100 (150:1 *v/v*) and considered as a positive control (I_∞)_. Fluorescence was measured by fluorometry at Ex. 479 nm / Em. 590 nm (Hitachi F-4500 Fluorescence Spectrophotometer). Dox release was determined as Dox (%) = (It − Io) / (I_∞_ − Io) × 100. Stability of liposomes was calculated as 100 – Dox (%).

### *In Vitro* Dox Release

Temperature-dependent Dox release kinetics from TSL and RGD-TSL were performed by fluorometry upon incubating the TSL samples (10 mM [lipid]) in pre-heated 90% fetal calf serum (FCS) (1:9 *v/v*) at temperatures ranging between 37 and 45°C for 5 min in a thermal-shaker (Eppendorf Thermomixer) at 300 rpm. Samples without incubation were considered as a blank (Io). After incubation, the samples were diluted in 10 mM Tris/NaCl 0.9%, pH 8.0 at 1:50 (*v/v*) and Dox fluorescence was measured by fluorimetry at Ex. 479 nm / Em. 590 nm. Maximum Dox fluorescence (positive control) (I_∞)_ was achieved when incubating TSL suspension (10 mM [lipid]) in 2% Triton X-100 in H_2_O for 30 min in a thermal shaker at 55°C and 1400 rpm. The Dox release (%) was calculated as Dox (%) = (It−Io) / (I_∞_−Io) × 100. *In vitro* time-dependent Dox release from TSL and RGD-TSL was measured at 42°C. TSL suspension (10 mM [lipid]) was mixed with pre-heated FCS (1:149 *v/v*) under stirring and Dox release was measured over time (at 1, 2, 3, 4, 5, 30, 60 min). TSL samples without heating were considered as a blank. TSL were destroyed by adding 10% Triton X-100 (150:1 *v/v*) and considered as a positive control. The Dox release (%) was calculated as described above.

### Cell Culture

Tumor cell lines B16Bl6 (murine melanoma) and B16F10 (metastatic murine melanoma) were cultured in a Dulbecco’s Modified Eagles’ medium (Lonza, Belgium) containing 10% FCS. Human umbilical vein endothelial cells (HUVECs) were isolated in-house and cultured in Human Endothelial SFM medium (Gibco, Invitrogen) enriched with 30% FCS. Cells were passaged once a week using Trypsin (Sigma, Aldrich) and maintained at 37°C, 5% CO_2_ in a humidified incubator. All experiments were performed at a confluence of 80–90%.

### *In Vitro* Dox-TSL Toxicity

B16Bl6, B16F10 and HUVEC cells were plated in 96 well plates at concentration 12,000 cells/well for B16Bl6 and B16F10 and 6000/well for HUVEC. The cells were allowed to attach for 24 h at 37°C and after that incubated with various concentrations of free Dox, Dox-TSL or RGD-Dox-TSL for 3 h at 37°C. After 3 h, liposomes were removed and cells washed three times with medium with FCS. Plates were placed at either normothermia (NT; 37°C) or hyperthermia (HT; water bath at 42°C for 1 h) and then left in the incubator at 37°C for additional 72 h. Cell survival was determined by XTT assay. Electron coupling reagent *N*-methyl dibenzopyrazine methylsulfate (1.25 mM in PBS; Sigma) (100 μl) was mixed with 5 ml of XTT solution (1 mg/ml in RPMI 1640) and each well was incubated with 50 μl of this mixture for 1 h at 37°C. Afterwards, XTT conversion was measured at 490 nm in a PerkinElmer Victor Wallac plate reader (Perkin Elmer, Groningen, The Netherlands).

### Flow Cytometry

Binding of TSL or RGD-TSL to B16Bl6, B16F10 and HUVEC cells was assessed by flow cytometry (FACS) analysis. 1 × 10^5^ cells in suspension were incubated for 3 h at 37°C with 400 nmol/ml TSL or RGD-TSL labelled with 0,3 mol% NBD-PE. After incubation, cells were washed three times with medium with FCS to remove unbound liposomes. Liposomal NBD-PE fluorescence was determined at excitation and emission wavelengths of 470 and 530 nm by a BD FACScan (Becton Dickinson, San Jose, CA, USA). Dead cells were labelled with propidiumiodide (PI) (Sigma, Aldrich) and 10,000 gated events were acquired per sample and samples were prepared in triplicate. For Dox uptake FACS analysis, the same amount of cells was incubated with 100 μM Dox for 3 h and subsequently washed 3× with medium with FCS. Cells in suspension were placed either at normothermia (37°C) or hyperthermia (42°C) for 1 h. Dox fluorescence was determined at excitation and emission wavelengths of 488 and 585 nm, respectively. As a dead cell marker, Sytox Green (Invitrogen) was used. Data was analysed with FlowJo software. Experiments were performed three times with three different batches of liposomes.

### Live Cell Confocal Microscopy

B16Bl6, B16F10 or HUVEC cells were seeded at the same concentrations as for fluorescent microscopy in cell culture chambers containing a cover glass insert coated with 0.1% gelatine. Cells were allowed to recover for 24 h. After 24 h, cells were incubated with 400 nmol/ml NBD-PE labelled TSL or RGD-TSL for 3 h at 37°C and for 30 min with lysotracker (LysoTracker® Red DND-99). After incubation, cells were washed three times with DMEM (B16Bl6 and B16F10) or HUVEC medium (HUVEC). Cells were analyzed on a Zeiss LSM 510 META confocal laser scanning microscope. NBD-PE fluorescence was detected by 513 nm argon laser and lysotracker was monitored by a 543 nm Helium –Neon laser. For Dox release experiments, cells were incubated with NBD-PE or DiD labelled Dox-TSL or RGD-Dox-TSL for 3 h at 37°C and after that washed three times with medium with FCS. Dox release was followed in time for 1 h at 42°C (40 × objective lens, 2,5 μm pinhole*)* and its fluorescence was detected by a 543 nm Helium –Neon laser. Images of 1024 × 1024 pixels were analyzed using Zeiss LSM image software (Zeiss, Germany).

### Animal Models

B16Bl6 (murine melanoma) cells were cultured in DMEM medium with 10% FCS. Ten million tumor cells were injected subcutaneously in the flanks of C57Bl6 mice and bulk tumors of 10 mm in diameter were used for transplantation into C57Bl6, expressing an eNOS-tag-GFP fusion protein constitutively in their vascular endothelium. Tumor pieces were implanted in a dorsal skin flap window chamber for intravital imaging. Bulk mice were housed at 20–22°C, humidity of 50–60%. Window chamber-bearing mice were used for experiments after 8–12 days of tumor implantation when tumor size reached 4–6 mm in diameter. These mice were housed in an incubator room with a humidity of 70% and temperature of 30–32°C. All mice were fed a standard laboratory diet *ad libitum* (Hope Farms Woerden, the Netherlands). Mice weighing 20–25 g were used for experiments. All animal experiments were performed in compliance with protocols approved by the committee on Animal Research of the Erasmus MC, Rotterdam, The Netherlands.

### Intravital Microscopy for Dox Release upon Hyperthermia and Uptake by Tumor and Angiogenic Endothelial Cells

Liposome binding to tumor vasculature and their clearance from circulation was analysed by intravital microscopy after injection of DiD-labelled TSL or RGD-TSL and followed up to 24 or 5 h respectively. In order to evaluate Dox release during hyperthermia and its uptake by tumor vascular endothelial cells and tumor cells, DiD-labelled Dox-TSL or RGD-Dox-TSL were injected i.v. through the penile or tail vein at a dose of 5 mg/kgDox. Both formulations were allowed to circulate for 5 h at body temperature in order to be able to bind to vascular angiogenic endothelial cells or tumor cells and observed by confocal microscopy (Zeiss LSM 510 META). After 5 h of circulation, tumor was heated at 42°C for 1 h and Dox release and uptake was detected as above (20× objective lens). Regions of interest were selected before, during and in the end of the hyperthermia treatment. Images of 1024 × 1024 pixels were analyzed using Zeiss LSM image software (Zeiss, Germany).

### *In Vivo* Dox Quantification

The integrated density (IntDen) from the red channel (obtained after setting a threshold) representing released doxorubicin at 42°C into mice injected with either RGD-Dox-TSL or Dox-TSL was quantified from 13 positions from each group, which were obtained from three mice. The data are presented as an average of IntDen of all the positions of each mouse. The data was analyzed by Image J software. When DiD quantified, 3–6 positions were used per mouse obtained from three mice from each group. The data are presented as an average of IntDen of all the positions of each mouse.

### Pharmacokinetic and Biodistribution of Dox-TSL and Dox-RGD-TSL

Pharmacokinetic and biodistribution of Dox-TSL and Dox-RGD-TSL was followed in B16Bl6 tumor bearing mice upon NT or initial HT conditions. At NT condition, mice were injected with 3 mg/kg Dox and blood sampling was performed at 0.1;1;2;4;6 and 24 h and organs were collected 24 h after liposome injection. At HT condition, tumors were first preheated for 1 h at 41°C and then cooled down for 15 min, in order to facilitate liposome extravasation. Then, liposomes were injected at 3 mg/kg Dox and blood samples were collected up to 24 h (0.1;1;2;6;24 h), after which the organs were removed. The Dox concentration in the blood and organs was analyzed by HPLC and calculated as% injected dose/g tissue (%ID/g). Six mice were used per each group.

### Statistics

*In vivo* drug release was analyzed by Mann-Whitney test and results with *p*-value ≤ 0.05 were considered statistically significant.

## Results

### Liposome Characterization

**Fig. 1:**
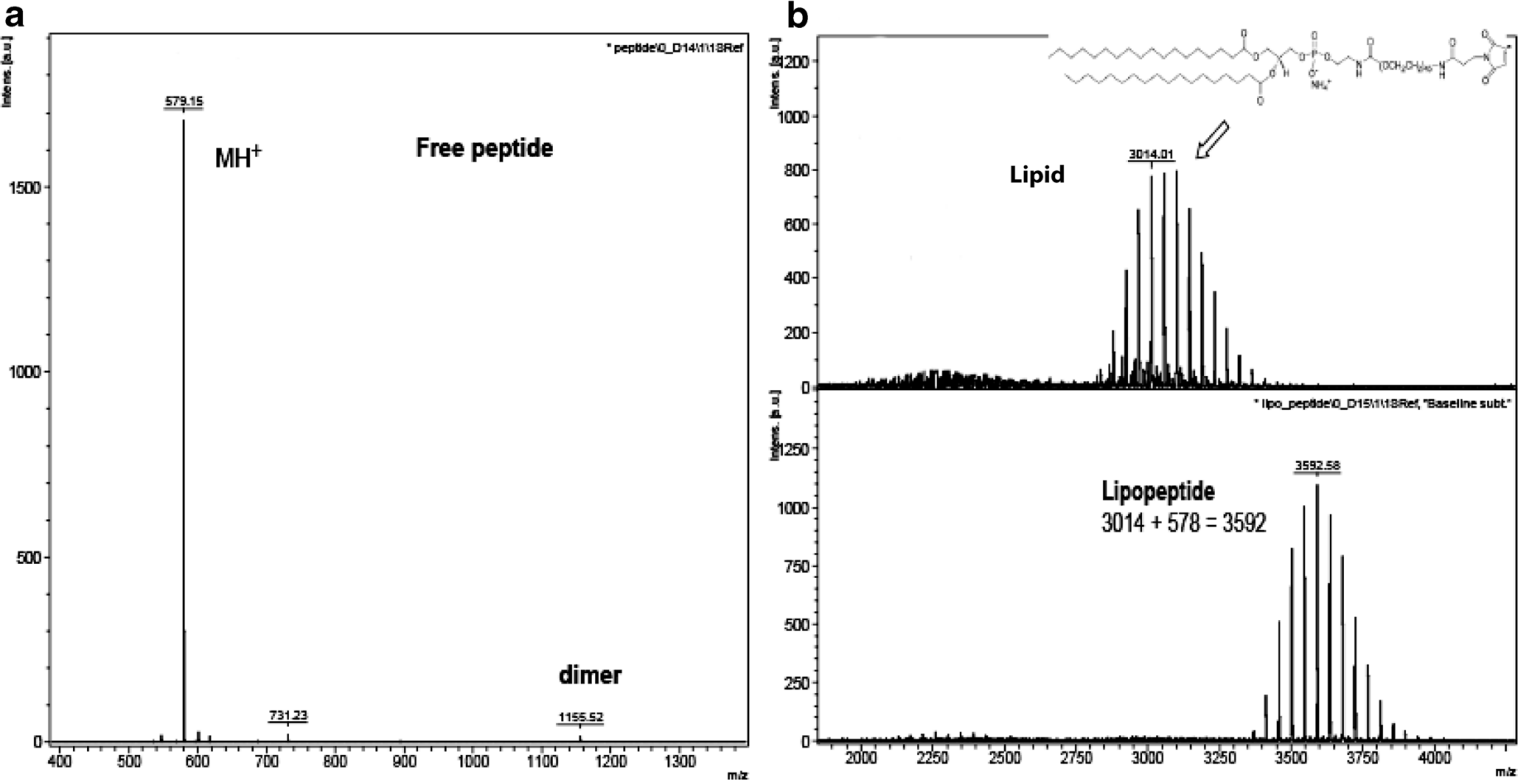
MALDI-TOFF spectra of the free peptide RGD (**a**), the free lipid (**b** *upper panel*) and the coupled RGD to mPEG in lipopeptide (**b** *lower panel*).

RGD-TSL and TSL consisted of the phospholipids DPPC, DSPC, DSPE-PEG2000 in a molar ratio (70:25:5). In the RGD-TSL formulation, RGD peptide was coupled to mPEG2000 and its coupling efficiency was analyzed by MALDI (Fig. 1), after which it was mixed with all the lipids in a molar ratio (70:25:5). Both formulations were prepared by lipid film hydration and extrusion method (28). Dox was loaded into the liposomes after extrusion by (NH_4_)_2_SO_4_ loading method (29). Liposomes were characterized by measuring size, polydisperity index (pdI), encapsulation efficiency and stability. All liposomes were ~85 nm after extrusion and with a pdI < 0.1. After Dox loading, liposomes retained their small size and a low pdI. Encapsulation efficiency of both formulations was >95%. Liposomes were comparably stable after 1 h of incubation at 37°C in 99% FCS. TSL contained 92% ± 1.8 of the entrapped Dox after 1 h at 37°C whereas RGD-TSL contained 85% ± 2.3 (data not shown).

### Temperature- and Time-Dependent Dox Release

**Fig. 2:**
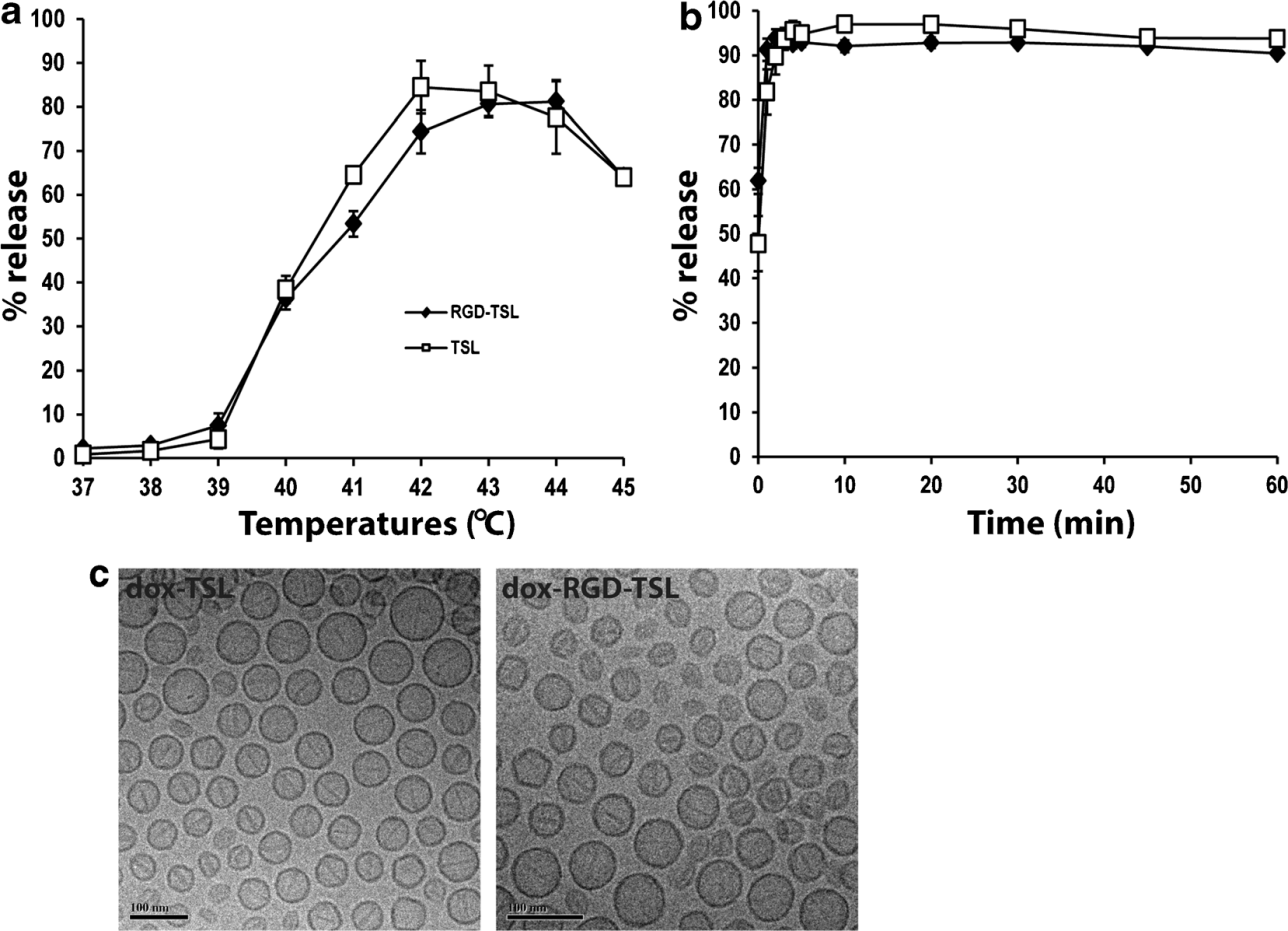
Temperature- (**a**) and time- (**b**) dependent Dox release kinetics from RGD-TSL and TSL. Temperature-dependent drug release profile was performed in temperatures between 37 and 45°C for 5 min in 90% FCS. Time-dependent release was carried for 1 h at 42°C in 99,7% FCS. Mean of three independent experiments with three different batches of liposomes. TEM-cryo imaging (**c**) of Dox-TSL and Dox-RGD-TSL. Bar, 100 nm.

In order to test whether liposomes are thermosensitive, their temperature- and time-dependent drug release profiles were determined (Fig. 2a and b). According to the temperature-dependent release kinetics (A), which was performed at varying temperatures (37–45°C), both formulations were thermosensitive showing an increasing drug release with increasing temperature. The formulations were stable at physiological temperatures (37–38°C) in 5 min and started to release their drug payload slightly at 39°C. The maximum drug release from TSL in 5 min was observed at 42°C (~85%), whereas for RGD-TSL it was at 43–44°C (~80%), after which temperatures the drug release declined as seen before (21).

Time-dependent release profile at a constant temperature of 42°C for 1 h was similar for both formulations (Fig. 2b). RGD-TSL and TSL rapidly released Dox in the first minute and released >90% of the encapsulated Dox in 1 h.

Active loading of Dox into RGD-TSL and TSL by ammonium sulphate gradient method resulted in an abundant amount of intraliposomal Dox crystals, which were well visualized by TEM cryo imaging in both formulations (Fig. 2c).

### RGD-TSL Demonstrate Higher Uptake by Tumor and Endothelial Cells than Non-targeted TSL

**Fig. 3:**
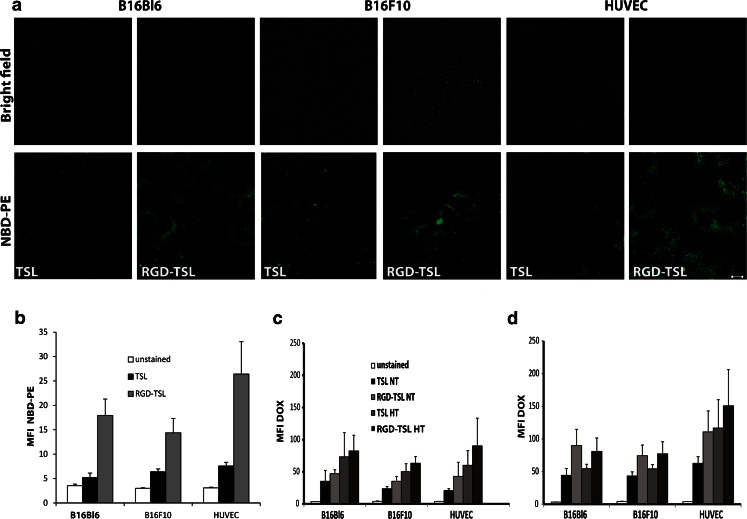
Confocal live-cell imaging for preferential uptake of RGD-TSL compared to TSL into B16Bl6, B16F10 and HUVEC cells after 3 h of incubation at 37°C (**a**). Unbound liposomes were removed by washing. Scale bar applies for all images, 10 μm. Intracellular NBD-PE fluorescent intensity represented as mean fluorescent intensity (MFI) in melanoma B16Bl6, B16F10 cells and HUVEC (**b**) treated with either TSL or RGD-TSL for 3 h at 37°C. Unbound liposomes were removed by washing. As unstained cells are used cells which were not incubated with liposomes. **c** and **d**. Intracellular Dox uptake represented as MFI in melanoma B16Bl6, B16F10 cells and HUVEC after 1 h (**c**) or 3 h (**d**) of incubation at 37°C, washing of unbound liposomes, followed by 1 h of HT at 42°C.

In order to test whether RGD coupling to liposomes increases liposomal uptake into tumor and endothelial cells, confocal microscopy and FACS analysis on melanoma B16Bl6 and B16F10 cells and HUVEC with NBD-PE labelled TSL or RGD-TSL was performed. Confocal microscopy on B16Bl6, B16F10 and HUVEC demonstrated that RGD-TSL are more abundantly taken up by the tumor cells and HUVEC compared to TSL (Fig. 3a). The 3 h incubation period at 37°C followed by a washing step for removal of unbound liposomes revealed that the RGD modification of TSL led to a preferential binding and uptake by all the cell lines. This was confirmed also by FACS analysis (Fig. 3b). In order to measure Dox delivery, FACS analysis was performed in B16Bl6, B16F10 and HUVEC after 1 h (C) or 3 h (D) of incubation with Dox-TSL or Dox-RGD-TSL followed by 1 h of HT at 42°C. The amount of Dox delivered from RGD-TSL at NT was higher than TSL in all cell lines and this amount further increased after 3 h of incubation. When HT was applied to the cells after 1 h of incubation at 37°C, there was an increase in Dox release and uptake in all cell lines. HT trigger did not further increase the delivered Dox to B16Bl6 and B16F10 cells after 3 h of incubation. Only in HUVECs there was an increased delivery of Dox upon HT after 3 h of incubation.

### Uptake of RGD-TSL in Lysosomes and Intracellular, HT-Triggered Dox Release from RGD-TSL in Tumor and Endothelial Cells

**Fig. 4:**
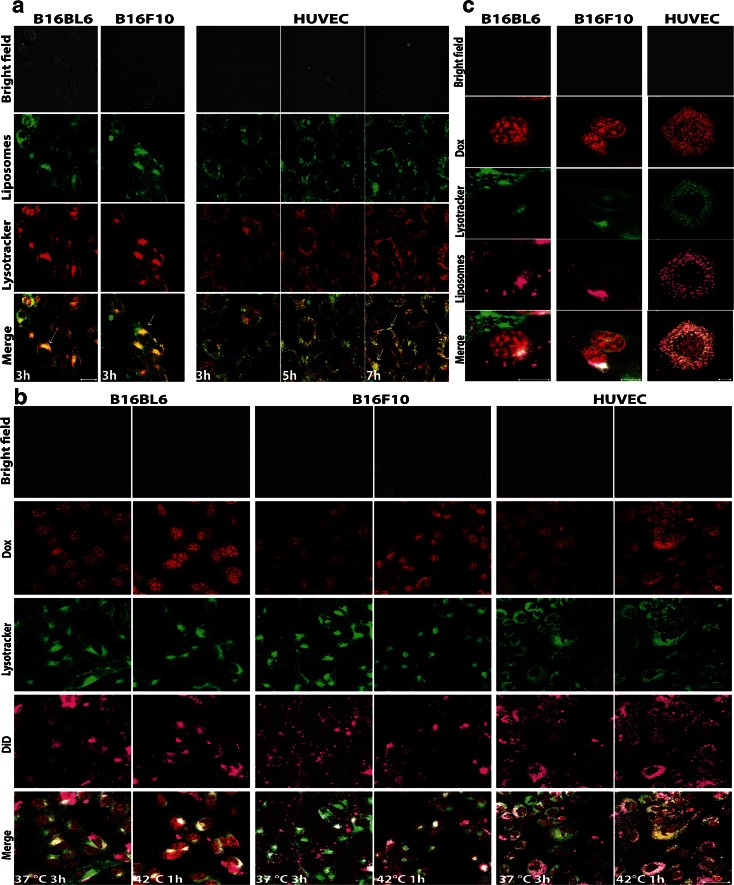
(**a**). Confocal microscopy on melanoma B16Bl6 and B16F10 cells and HUVEC incubated with NBD-PE (*green*) labelled RGD-TSL for 3 h at 37°C and lysotracker (*red*). Unbound liposomes were removed by washing 3× with medium without FCS. After washing, B16Bl6 and B16F10 were immediately imaged at 37°C, whereas HUVEC were followed up to 7 h. Internalized liposomes in the cytosol can be observed in all the cell lines (*white arrows*). Non-internalized in the lysosomes liposomes were also visible (*blue arrows*). B16Bl6 and B16 internalized liposomes immediately in the lysosomes after the 3 h of incubation period (*yellow* colocalization of green liposomes and red lysotracker), whereas this process happened in HUVEC after 7 h. Images were taken by confocal microscope (40×, 2,5 μm pinhole, 2× zoom). Scale bar applies for all images, 20 μm. (**b**). Doxorubicin release (*red*) from DiD-labelled RGD-TSL (*purple*) in B16Bl6, B16F10 and HUVEC upon HT trigger. Cells were incubated with 50 μM Dox for 3 h at 37°C, after which cells were washed 3× with medium without FCS. Images were taken right after this incubation at 37°C. Then, HT at 42°C for 1 h was applied and images in the end of the HT treatment were recorded. Images were taken by confocal microscope. Scale bar applies for all images, 50 μm. (**c**). Colocalization of RGD-TSL (*purple*) with lysotracker (*green*) and Dox release (*red*) in the lysosomes. Scale bar applies for all images, 10 μm.

The intracellular localization of liposomes was studied by live cell imaging in B16Bl6, B16F10 and HUVEC (Fig. 4a). Cells were incubated with NBD-PE labelled (green) RGD-TSL and lysotracker (red, to stain lysosomes) for 3 h at 37°C, after which the unbound liposomes were removed by washing. B16Bl6 and B16F10 were able to localize the RGD-TSL in the cytoplasm after 3 h of incubation and those liposomes were colocalized with the red lysotracker, seen as yellow fluorescence signal (white arrows). Still, there were some RGD-TSL (green fluorescence spots), which were not concentrated in the acidic compartments (blue arrows). By contrast, in HUVEC sequestering of RGD-TSL in the lysosomes occurred at a slower pace. After 3 h of incubation at 37°C, liposomes and lysotracker were observed as green and red separated fluorescence signals, showing no entrapment of the liposomes in the lysosomes. However, when cells were followed for a prolonged period, colocalization (in yellow) started to be visible. After 7 h of incubation, there was an abundant amount of RGD-TSL localized in the lysosomes but also some non-entrapped liposomes in lysosomes could be observed.

To prove that Dox-RGD-TSL deliver the encapsulated drug intracellular, live cell imaging on B16Bl6, B16F10 and HUVEC was performed using DiD labelled Dox-RGD-TSL and green lysotracker (Fig. 4b). Removing the unbound liposomes 3 h after incubation at 37°C resulted in some doxorubicin release inside of all cell lines, most probably due to processing of liposomes by the cells in this time frame. The released Dox localized mainly in the cell nuclei but could also be observed in the cytoplasm. The application of HT for 1 h triggered additional Dox release, seen as increased red fluorescence signal intracellularly. In B16Bl6 and B16F10, the Dox delivery upon HT treatment was predominantly nuclear, whereas in HUVEC it was cytoplasmic (Fig. 4b and c). In accordance with Fig. 4a, DiD-labeled RGD-TSL (purple) also localized in lysosomes (green) to some extent. Non-colocalized liposomes with lysosomes could also be observed. Interestingly, the released cytoplasmic Dox upon HT colocalized with the green lysotracker, which indicates Dox release from RGD-TSL also occurs in acidic compartments in the cytosol (Fig. 4b and c).

### Cytotoxicity of Dox Encapsulated in TSL and RGD-TSL upon NT and HT

**Fig. 5:**
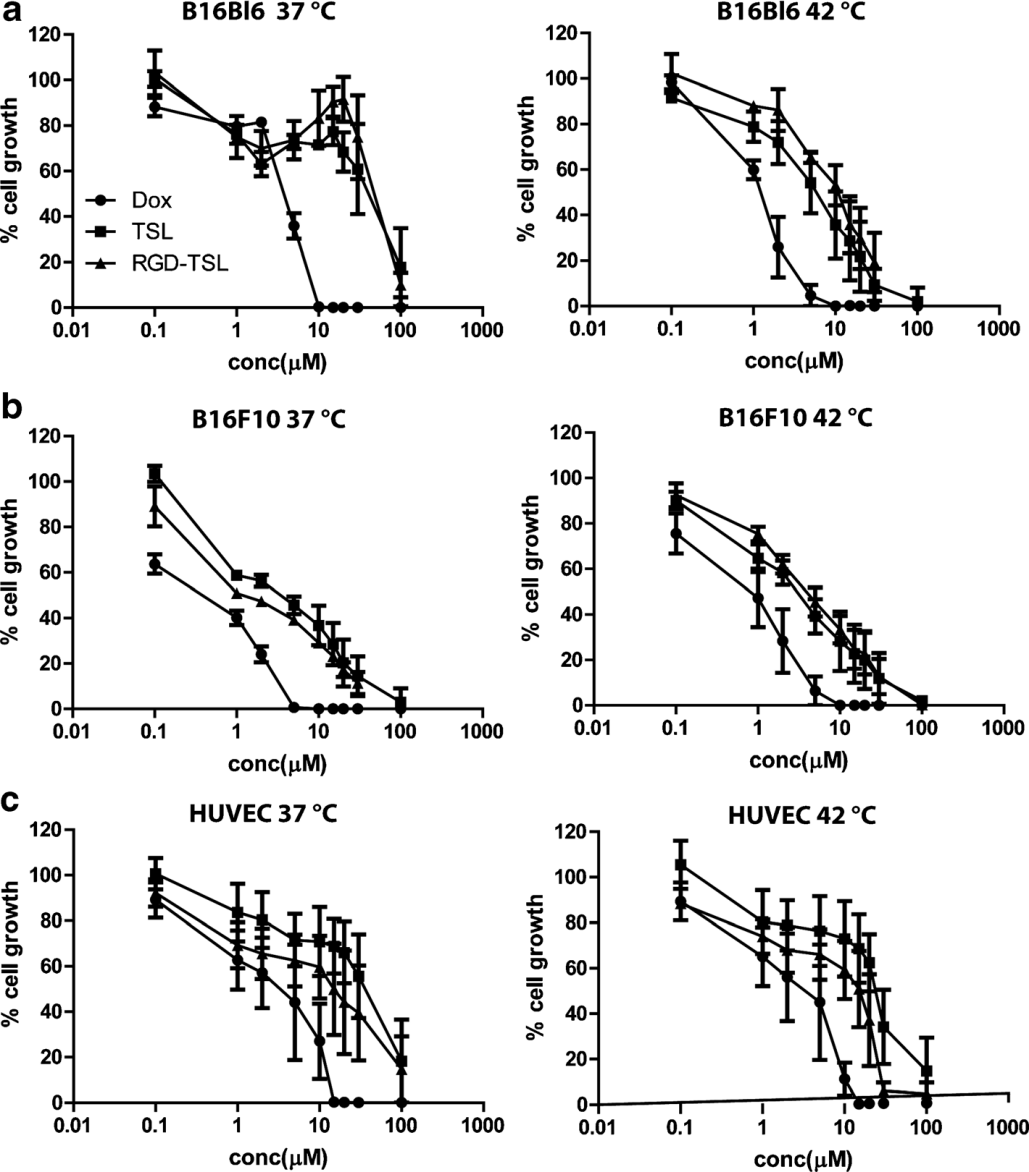
Cytotoxicity of Dox-TSL and Dox-RGD-TSL either at 37 or 42°C in B16Bl6 (**a**), B16F10 (**b**) and HUVEC (**c**) determined 72 h after liposome incubation.

Incubation of B16Bl6,B16F10 and HUVEC cells for 3 h with Dox-TSL or Dox-RGD-TSL showed no differences in cell toxicity determined 72 h later as seen in Fig. 5 and Table 1. Application of HT for 1 h at 42°C after 3 h of liposomes incubation could not further improve cytotoxicity impact 72 h later in B16F10 and HUVEC but only to some extent in B16Bl6 cells. Free Dox demonstrated the highest cytotoxicity on all the cell lines because it is rapidly taken up by the cells in its free form.

**Table 1:**
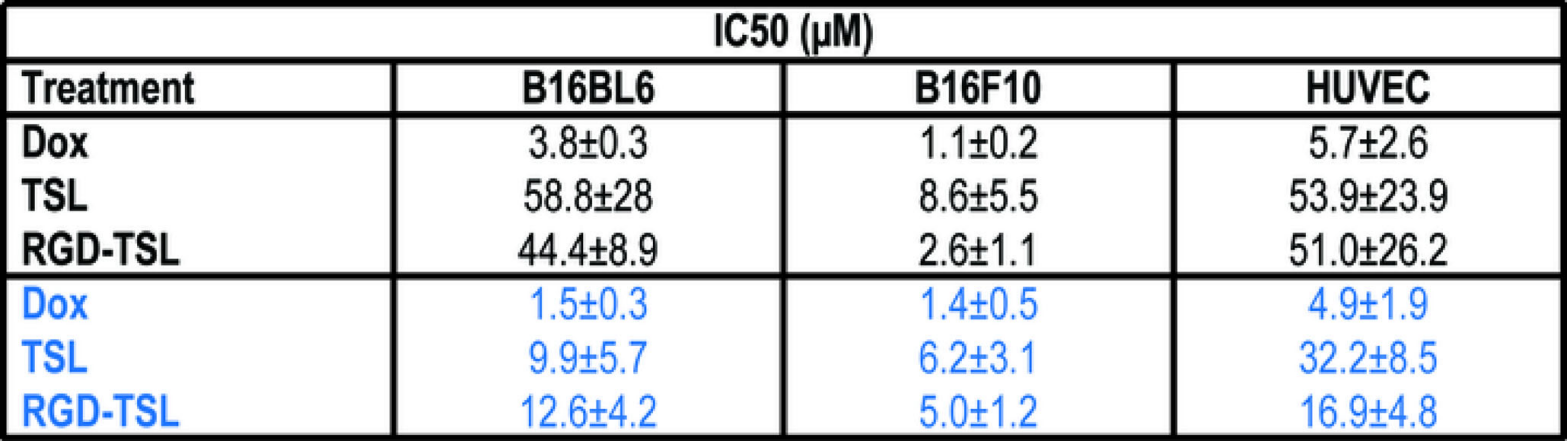
*IC 50 Values in μM Dox of B16Bl6 Murine Melanoma, B16F10 Murine Melanoma and Endothelial Cell (HUVEC) Treated with Free Dox or Dox Formulated in TSL or RGD-TSL for 1 h at 37°C. After removal of unbound liposomes, cells were subjected to HT at 42°C for another 1 h (in blue) or NT (in black) as a control

### Binding to- and Extravasation from Tumor Vasculature of TSL and RGD-TSL

**Fig. 6:**
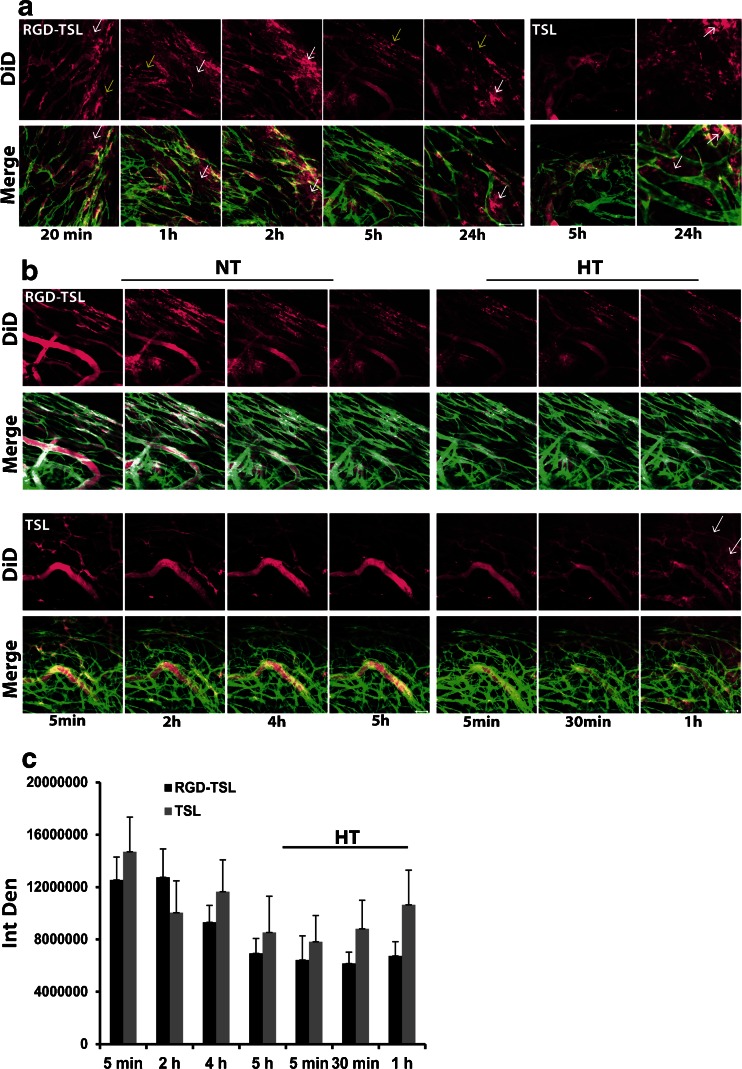
(**a**). Binding of DiD-labeled RGD-TSL (*purple*) to tumor vasculature (*green*) of B16Bl6 window chamber bearing mice. Binding of liposomes to tumor endothelial cells started 20 min after injection and was followed in time up to 24 h. Representative images from intravital microscopy were selected. Scale bar applies to all images, 50 μm. (**b**). RGD-TSL and TSL appearance (DiD, in *purple*) in tumor vasculature (*green*) during 5 h at NT in B16Bl6 window bearing mice (Fig B *left panels*) and upon subsequent HT at 42°C for 1 h (Fig. 5b *right panels*). DiD-labelled RGD-TSL or TSL were injected i.v, after which they were allowed to circulate in blood stream at NT for 5 h in order to allow binding of RGD-TSL to angiogenic endothelial cells. Thereafter, HT at 42°C for 1 h was applied to promote extravasation of RGD-TSL and TSL. Scale bar applies to all images 50 μm. (**c**). *In vivo* quantification of DiD liposomal fluorescence before and during 1 h of HT, presented as integrated density (IntDen) in time, see Materials and methods for details.

In order to proof that RGD-TSL target angiogenic endothelial cells *in vivo*, intravital microscopy in B16Bl6 window chamber bearing mice was performed. To visualize circulating liposomes in the blood stream, liposomes were labelled with DiD (purple). In these mice tumor vasculature is visualized by the constitutive expression of a GFP-eNOS-tag fusion protein in endothelial cells (Fig. 6). Twenty minutes after injection of RGD-TSL, next to circulating liposomes in the lumen of the blood vessels, bound RGD-TSL could be observed (Fig. 6a yellow arrows). These liposomes can be visualized as patchy fluorescent spots on the vessel walls. Besides bound liposomes to the angiogenic endothelial cells, extravasated liposomes from tumor vasculature are visible already 20 min after injection (white arrows). They are visible as diffuse purple fluorescence outside of the green blood vessels. Binding continued in time and was pronounced 24 h after injection. In contrast, using DiD-labeled TSL, no binding to tumor vasculature was detected after 5 h of circulation. In the last time point (24 h), only extravasated TSL were visible (Fig. 6a, right panel).

Liposome clearance from circulation during the 5 h targeting phase and Dox triggered release upon HT was further evaluated by intravital microscopy in B16Bl6 window chamber bearing mice (Fig. 6b). Liposome clearance was observed already 2 h after injection by decrease in DiD fluorescence signal from both RGD-TSL and TSL. Clearance of liposomes continued in time up to 5 h (Fig. 6b and c). However, RGD-TSL bound to tumor vasculature were visible already 5 min after injection, whereas this was not observed for TSL even after 5 h of circulation, which is in accordance with Fig. 6a. HT trigger at 42°C for 1 h did not seem to cause any additional clearance neither of RGD-TSL or TSL. Upon HT, TSL could be observed extravasated from the tumor vasculature (white arrows), a process which is known to be heterogeneous within the tumor. This is in accordance with the quantification of the images, showing an increase of the TSL signal upon HT, which is most probably due to extravasation. However, there was no increase of the RGD-TSL levels upon HT (Fig. 6c).

### *In Vivo* Dox Release upon HT and Dox Uptake by Tumor and Endothelial Cells

**Fig. 7:**
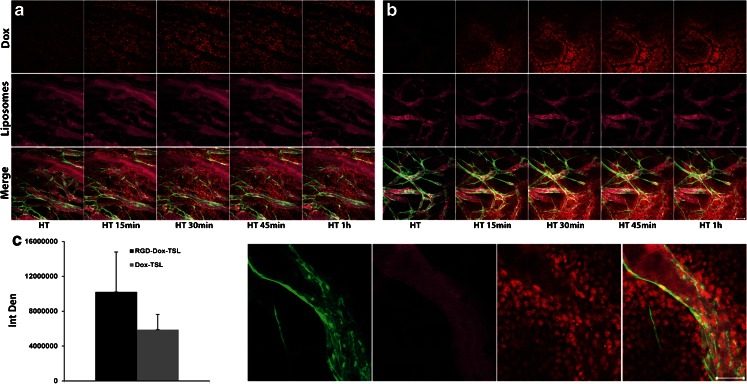
Dox release upon HT treatment from TSL (**a**) and RGD-TSL (**b**) in B16Bl6 window chamber bearing mice. Mice were injected with 5 mg/kg Dox in DiD-labelled (*purple*) TSL or RGD-TSL. After 5 h of liposome circulation, a temperature of 42°C for 1 h was applied to trigger Dox release. Representative images were taken from the beginning of the HT treatment up to 1 h. (**c**). *In vivo* quantification of Dox released from RGD-TSL or TSL 1 h after HT treatment, presented as integrated density (IntDen), see materials and methods. (**d**). Dox uptake in endothelial cells (*green*) and tumor cells from RGD-TSL after 1 h of HT treatment. Scale bar applies for all images, 50 μm.

In order to understand whether RGD-TSL release Dox *in vivo* upon HT treatment and to follow Dox distribution in the tumor, intravital microscopy was performed. Circulation of TSL and RGD-TSL for 5 h in the blood stream did not cause any premature release of Dox (data not shown). However, when HT at 42°C was applied, immediate Dox release (red) was observed from TSL (Fig. 7a) and RGD-TSL (Fig 7b). Dox from both formulations was released first intravascularly from the circulating DiD-labelled liposomes (purple), after which it was gradually taken up by endothelial cells and tumor cells surrounding the blood vessels (Fig. 7d and supporting information, video 1). The Dox uptake in the both treatment groups increased in time and was maximal after 1 h of HT, when also the lumen of the blood vessels was cleared from Dox. Quantification of the images showed that the amount of delivered Dox to the tumor from RGD-TSL was 1.7 fold higher than from TSL (Fig. 7c). However, this difference was not statistically significant (*p*-value = 0.8).

**Fig. 8:**
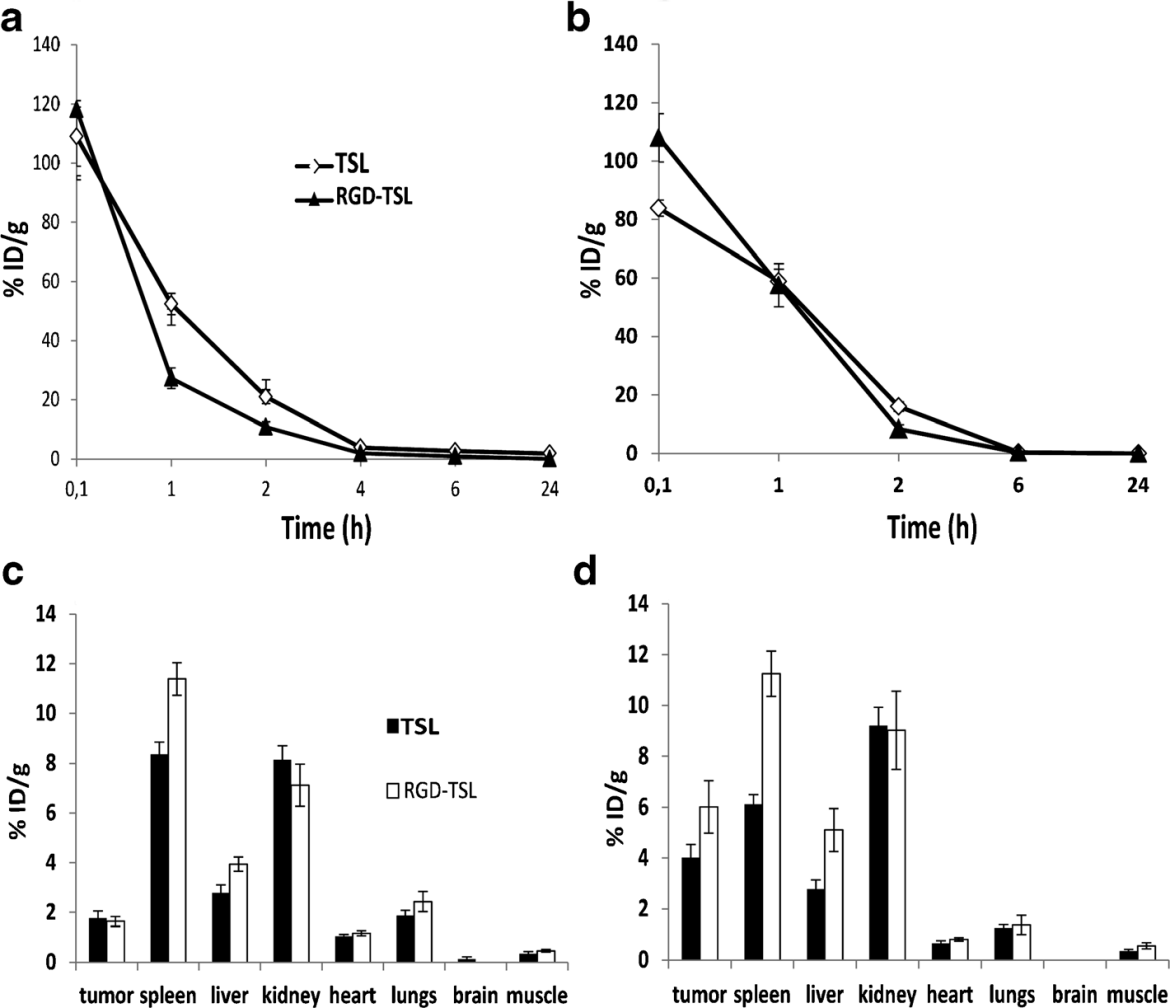
Pharmacokinetics (**a** and **b**) and biodistribution (**c** and **d**) of Dox-TSL and Dox-RGD-TSL in B16BL6 tumor bearing mice upon NT or initial HT conditions. A,C. At NT condition, mice were injected with 3 mg/kg Dox and blood sampling was performed at the indicated time points and organs collected 24 h after liposomes injection. At HT condition (**b** and **d**), tumors in mice were preheated for 1 h at 41°C and cooled down for 15 min, in order to allow for liposome extravasation. Then, liposomes were injected at 3 mg/kg Dox and blood samples were collected up to 24 h, after which the organs were removed. The Dox concentration in the blood and organs was analyzed by HPLC.

To follow up Dox clearance from circulation, its distribution in healthy organs and tumors and to be able to quantify Dox concentrations in tumors and organs, the pharmacokinetic and biodistribution profiles of Dox in TSL or RGD-TSL were studied (Fig. 8) under NT or initail HT conditions. At NT condition (Fig. 8a and c), Dox from both formulations seemed to clear from circulation quite fast in the first 1 h, as it was faster for RGD-TSL than for TSL (27% v/s 52% remaining Dox respectively). After 2 h of liposome circulation, the trend was the same showing lower remaining Dox from RGD-TSL than from TSL (10% v/s 20% respectively). At later time points (4,6,24 h) there was barely any Dox present in circulation from TSL and RGD-TSL. The application of initial HT (Fig. 8b) seemed to increase the presence of Dox from RGD-TSL in circulation at 1 h time point, after which its clearance was the same as at NT conditions. Besides, upon initial HT conditions, clearance of Dox from TSL and RGD-TSL was similar. Considering the biodistribution of Dox (Fig. 8c and d), at both NT and HT conditions, there was a significant uptake of Dox from both formulations in the spleen as it was higher for Dox from RGD-TSL than TSL (11.3 respectively v/s 8.3% ID/g at NT; and 11 v/s 6% ID/g at HT). Similar high Dox accumulation in the kidney was observed from both formulations. Dox accumulated in the liver was similar for RGD-TSL and TSL under NT (3.9%ID/g respectively v/s 2.7%ID/g). The higher Dox uptake in spleen and liver from RGD-TSL is due to most probably opsonization of RGD-TSL by proteins in these organs. There was a minimal uptake of Dox from TSL and RGD-TSL in the heart, lungs and muscle upon NT and HT. No Dox was detected in the brain from neither of the formulations. At NT, the tumor uptake of Dox was similar for both formulations. Application of initial HT for 1 h at 41°C was able to cause ~ 3.7 fold increase of Dox delivery to the tumor from RGD-TSL (1.6 v/s 6% ID/g) and ~ 2.3 fold increased Dox amount to the tumor from TSL (1.7 v/s 4% ID/g). The amount of Dox delivered to the tumor upon initial HT conditions from RGD-TSL was not significantly different from Dox delivered from TSL (*p*-value 0.1).

## Discussion

Nanoparticles, such as liposomes have been successfully designed and used in treatment of various types of cancer (30). Although modifications to these nanocarriers contributed to decreased drug-related side effects, their high stability (31–33) and limited tumor localization (34) prevent the desired increase in therapeutic outcome (4, 7). Forcing encapsulated drug to leave the liposome, as is achieved with TSL exposed to hyperthermia, leads to an enhanced drug release locally in the tumor (35–37). Also, application of an external trigger such as HT, can promote liposome extravasation from tumor vasculature and increase their accumulation locally in the tumor area (18–20, 38). When combined, this interstitial release approach relies primarily on HT-augmented liposome extravasation followed by heat-triggered drug release (39). More directly, improved drug accumulation is observed with the intravascular release approach, for instance with lysolipid-thermosensitive liposomes (40). Next to the use of an external trigger for controlling drug release, decorating liposomes with targeting ligands specific for the tumor cells or vasculature can also increase the liposomal drug efficacy. We have recently developed cationic thermosensitive liposomes, a nanoparticle combining targeting and triggered release properties in one carrier (41, 42) and redesigned it in ordered to achieve a pharmaceutically stable formulation. In the present study, we report on the development of TSL decorated with another targeting ligand, cRGD (RGDf[N-Met]C, which is specific for integrins overexpressed on both tumor vasculature and tumor cells.

Engrafting of TSL with cRGD did not cause significant changes in the pharmaceutical properties of the formulation. Dox-TSL and Dox-RGD-TSL were similar in size, pdI, Dox encapsulation and Dox release kinetics. This is in accordance with Al-Ahmady *et al.* using monoclonal antibody-targeted thermosensitive liposomes showing that traditional thermosensitive liposomes maintain their physicochemical and thermal properties when conjugated to the monoclonal antibody (43). Both formulations showed to be stable at temperatures up to 38°C, slightly released Dox at 39°C and at 42°C released >90% of the encapsulated Dox in 1 h,characteristics favorable for use in clinical settings. In accordance with Kim *et al.* (44) and Al-Ahmady *et al.* (43) there was a preferential uptake of RGD-TSL by both melanoma and endothelial cells, which was confirmed by FACS analysis and confocal microscopy (Fig. 3a and b). The specificity of RGD-TSL for the tested cell lines contributed to an increased Dox delivery to all the cell lines at both NT and HT conditions (Fig. 3c and d). After 1 h of incubation at NT with either TSL or RGD-TSL, there was an increased Dox uptake by all the cell lines from RGD-TSL. HT additionally triggered the drug release and uptake from TSL and RGD-TSL, which was higher for RGD-TSL than TSL. In accordance with Al-Ahmady *et al.* (43), the longer incubation period of 3 h at 37°C led to an increased Dox uptake in the cell lines and was higher again for RGD-TSL. However, the subsequent HT trigger did not change the drug uptake in the melanoma cell lines, which is most likely due to the fact that in the 3 h period the cells are able to degrade the liposomes themselves preventing an additional release upon HT. Only an increase in drug uptake upon HT after 3 h of incubation was observed with HUVECs, which might be due to the fact that these cells take longer time to entrap liposomes in acidic compartments in the cytosol (Fig. 4a). In contrast, B16Bl6 and B16F10 were able to process endocytosed RGD-TSL in a faster manner.

Using confocal microscopy, we demonstrated that HT was able to trigger drug release and uptake *in vitro* in melanoma and endothelial cells. After 3 h of incubation of the cells at 37°C with Dox-RGD-TSL, there was a premature drug release and uptake most likely due to cellular processing of the drug at this time point. However, HT additionally increased Dox delivery in all the cell lines and was mostly nuclear for melanoma cells and both nuclear and cytoplasmic for HUVEC (Fig. 4b and c), which is in accordance with Kim *et al.* (44). There was no difference in cytotoxicity of Dox-TSL and Dox-RGD-TSL to any of the cell lines at NT conditions.

A HT trigger could not further increase the cytotoxicity of Dox-RGD-TSL in B16F10 and HUVEC but only in B16Bl6. This is likely due to the nature of the assay in which cytotoxicity is measured 72 h after nanoparticle incubation and heat treatment. During the remaining 72 h also non-heat triggered RGD-TSL will release their Dox contents intracellularly due to nanoparticle processing, causing cellular cytotoxicity.

The 5 h targeting phase did not show any premature Dox release from neither RGD-TSL or TSL (data not shown), which is in accordance with other targeted TSL (42). During this phase, RGD-TSL bound to tumor endothelial cells already 20 min after liposome injection and were observed bound up to 24 h. In contrast, TSL did not bind to tumor vasculature and were mostly extravasated 24 h after injection (Fig. 6a), which confirmed the targeting properties of RGD-TSL. Liposomes were cleared from circulation gradually in 5 h, shown by intravital microscopy and image quantification. The application of HT could increase TSL extravasation starting at 30 min of HT and increasing up to 1 h, which is in accordance with Li *et al.* (20) and Dicheva *et al.* (42) (Fig. 6b and c).

HT at 42°C could trigger a massive Dox release from both RGD-TSL and TSL. Release started already 5 min after heat trigger and increased up to 1 h (Fig. 7a). Image quantification from numerous positions in the tumor in several mice demonstrated that due to the RGD-TSL specificity for the tumor, these liposomes delivered 1.7 fold higher amount of doxorubicin than TSL (Fig. 7c). However, this difference was not statistically significant. Additionally, hyperthermia triggered release of Dox and Dox was subsequently taken up by both and angiogenic endothelial cells, which proves the dual targeting approach (Fig. 7d).

The pharmacokinetic behavior of Dox-TSL and Dox-RGD-TSL was investigated with or without initial HT. Initial HT was used to increase liposome extravasation. Dox from RGD-TSL was cleared faster from circulation than Dox from TSL under NT. This observation might show that targeting influenced Dox clearance. However, upon HT conditions, Dox clearance from circulation from the two formulations was similar.

Biodistribution studies showed that the highest uptake of Dox-TSL and Dox-RGD-TSL was in the spleen and the kidneys followed by the liver (Fig. 8c and d). The high spleen and liver uptake are due to the fact that these organs are part of the mononuclear phagocyte system (MPS), which is responsible for filtering out of liposomes from the blood circulation (45). There was no explanation why kidneys had an increased Dox uptake at both NT and HT conditions. As expected, there was a little Dox uptake from the two formulations under NT and HT in heart and lungs and no uptake in brain and the leg muscle close to the heated tumor. The absence of Dox in the leg muscle shows that the heating was restricted only to the tumor. There was not a difference in tumor uptake of Dox under NT by any of the formulations showing that targeting do not contribute to increased drug uptake at this conditions. However, when initial HT for 1 h at 41°C was applied, there was an increased Dox uptake in the tumor from both formulations, which is likely due to increased extravasation of liposomes upon HT and therefore their higher accumulation at the tumor site.

Future experiments will focus on improving the HT treatment protocols and will address the efficacy of our dual targeted and triggered drug delivery approach using cRGD thermosensitive liposomes.

## Conclusion

RGD-TSL encapsulating Dox were successfully developed with both targeting and triggered properties. They demonstrated specificity to tumor and endothelial cells as compared to non-targeted TSL. RGD-TSL were taken up by acidic compartments in cytosol and intracellularly released Dox upon HT. *In vivo*, RGD-TSL bound to angiogenic endothelial cells and massively released Dox when HT was applied. Biodistribution studies showed that initial HT treatment increases Dox delivery to the tumor from both formulations. Further studies will address the efficacy of our dual targeted and triggered Dox delivery approach using RGD-TSL.

## Electronic supplementary material

ESM 1 (AVI 16216 kb)

## Abbreviations

°C: Degree celsius
DMEM: Dulbecco modified eagle medium
Dox: Doxorubicin
DPPC: 1,2-dipalmitoyl-sn-glycero-3-phosphatidylcholine
DSPC: 1,2-distearoyl-sn-glycero-3- phosphatidylcholine
DSPE-PEG2000: 1,2-distearoyl-sn-glycero-3-phosphoethanolamine-N-PEG2000
Em.: Emission
Ex.: Excitation
FCS: Fetal calf serum
Fig.: Figure
GFP: Green fluorescence protein
H: Hours
HEPES: 4-(2-hydroxyethyl)-1-piperazineethanesulfonic acid
HT: Hyperthermia
i.v.: Intravenous
Min: Minutes
ml: Millilitre
NT: Normothermia
PDI: Polydisperisty index
S.c.: Subcutaneous
SD: Standard deviation
SEM: Standard error
TEM: Transmission electron microscopy
Tm: Melting temperature
TSL: Thermosensitive liposomes
